# The Medical Institutions of Calcutta

**Published:** 1899-04-15

**Authors:** 


### THE MEDICAL INSTITUTIONS OF CALCUTTA.

Although many improvements in the treatment of disease have been introduced in recent years, owing to greater skill on the part of the doctors and to a wider knowledge of the properties of drugs, it does not, according to the most recent reports on the Calcutta medical institutions, appear that any advance has been made in the methods of dealing with cholera. During 1897, though the disease was less prevalent in Calcutta itself than in the previous year, the admissions into the hospitals were a couple of hundred in excess of those of 1896. The increase was principally due to a large number of patients being brought into the Campbell Hospital from the Cooly depôts in Intally. But the most striking feature of the report is the great percentage of cases which still terminate fatally, in spite of all the efforts of doctors and nurses. Just ten years ago, 1887, the percentage of deaths amongst those treated for cholera in the hospitals was 55·7, in 1897 it was 55·33. It is true that between these dates the percentage has sometimes run higher—in 1891 and 1892, for instance, when it was 59·7 and 59·9 respectively, but the fact remains that during the last decade the treatment of cholera, so far as the saving of life is concerned, has been practically at a standstill. At two of the hospitals, the Medical College and the Howrah, the deaths were only 40 per cent. of the cases, but nothing of a satisfactory character can be argued from their statement, as the Principal of the former confesses that he cannot account for the improved returns in any way except that the attacks must have been of a milder type. The plan of treatment in every case was the same as the year before, when no such good result followed. The rate of mortality in the Presidency General Hospital is a high one—75 per cent.—but it is explained that this particular institution receives a great many seamen, who suffer from cholera of a very severe type, and it is notorious that a large proportion of the cases end disastrously. That dysentery and diarrhœa, which were both unusually fatal in 1897, are capable of treatment in a way which cannot be said of cholera is borne out by the small percentage of deaths amongst Europeans and Eurasians. Only 8 per cent. died from dysentery, 5 per cent. from diarrhœa in the Calcutta hospitals in 1897, whilst the loss amongst the native population was 35 and 44 per cent. respectively. But a considerable proportion of the patients who succumbed were really admitted in a moribund condition. Some had come, much emaciated, from the famine-stricken districts, and easily fell a victim to disease; others were pilgrims picked up by the roadside, and sent by the police to hospital in an almost dying state. At the time of the report the Dufferin Victoria Hospital did not seem to be doing so well as might be expected from it, the attendance during the past year having diminished by more than two thousand. But this was attributed by the lady superintendent to the unsuitability of the locality in which the hospital was situated, the limited accommodation in the temporary building, and the closure of the institution for a month for repairs. By now the permanent buildings are probably completed, and a change for the better will, it is hoped, be recorded. Lastly, it is gratifying to read of the praise given by the Lieutenant Governor upon the testimony of the medical officers to the trained nurses employed in the Calcutta Institution. The devotion of those at the Presidency General Hospital, under the superintendence of the Clewer Sisters, is especially spoken of in high terms. But the little rider which is added is decidedly amusing. "The superintendent has, however, noted a tendency on the part of the nurses to restrict their work to taking temperatures and administering medicines, relegating their other duties to menial servants. Steps have been taken to check this, and the whole question of nursing has been placed under the consideration of a committee."

